# Prognostic association of starvation-induced gene expression in head and neck cancer

**DOI:** 10.1038/s41598-021-98544-1

**Published:** 2021-09-27

**Authors:** Masakazu Hamada, Hiroaki Inaba, Kyoko Nishiyama, Sho Yoshida, Yoshiaki Yura, Michiyo Matsumoto-Nakano, Narikazu Uzawa

**Affiliations:** 1 Department of Oral and Maxillofacial Surgery II, Osaka University Graduate School of Dentistry, 1-8 Yamadaoka, Suita, 565-0871 Osaka, Japan; 2 Department of Pediatric Dentistry, Okayama University Graduate School of Medicine, Dentistry and Pharmaceutical Sciences, Okayama, Japan

**Keywords:** Head and neck cancer, Oral cancer

## Abstract

Autophagy-related genes (ARGs) have been implicated in the initiation and progression of malignant tumor promotion. To investigate the dynamics of expression of genes, including ARGs, head and neck squamous cell carcinoma (HNSCC) cells were placed under serum-free conditions to induce growth retardation and autophagy, and these starved cells were subjected to transcriptome analysis. Among the 21 starvation-induced genes (SIGs) located in the autophagy, cell proliferation, and survival signaling pathways, we identified SIGs that showed prominent up-regulation or down-regulation in vitro. These included AGR2, BST2, CALR, CD22, DDIT3, FOXA2, HSPA5, PIWIL4, PYCR1, SGK3, and TRIB3. The Cancer Genome Atlas (TCGA) database of HNSCC patients was used to examine the expression of up-regulated genes, and CALR, HSPA5, and TRIB3 were found to be highly expressed relative to solid normal tissue in cancer and the survival rate was reduced in patients with high expression. Protein–protein interaction analysis demonstrated the formation of a dense network of these genes. Cox regression analysis revealed that high expression of CALR, HSPA5, and TRIB3 was associated with poor prognosis in patients with TCGA-HNSCC. Therefore, these SIGs up-regulated under serum starvation may be molecular prognostic markers in HNSCC patients.

## Introduction

Head and neck cancer is the sixth most common malignancy in the world, 90–95% of which is squamous cell carcinoma (SCC). Over 60% of patients already have advanced cancer at the time of their first visit, with an estimated 5-year survival rate of 40–50%^1–4^. Surgery, radiation, chemotherapy, and targeted therapies are used to treat head and neck squamous cell carcinoma (HNSCC)^5,6^. In recent years, immunotherapy with antibodies that target the immune checkpoint pathway has been introduced and has shown long-term effects on cisplatin-resistant cancer, distant metastases, and recurrence of poor prognosis^7,8^. However, valid cases are limited to 18–25% of advanced HNSCCs^9^. Long-term immunological side effects are also a problem^10^. Effective indications for these therapies need to be searched and new therapies need to be developed.

A major advance in recent HNSCC research is the aggregation of extensive genetic analysis results of HNSCC^11–15^. A typical HNSCC database is the Cancer Genome Atlas (TCGA), published in 2015. Recent technological advances have enabled TCGA and other large-scale genomics studies to determine the broader landscape and frequency of chromosomal alterations, mutations, and expressed genes that contribute to HNSCC pathogenesis, prognosis, and resistance to therapy^11–15^. The TCGA-HNSCC database may be used to screen for differentially expressed genes (DEGs) in cancer and normal tissue transcriptome studies in HNSCC patients^16,17^. Furthermore, using the TCGA database, many studies on the deviation of genes and signaling pathways involved in carcinogenesis and prognosis are being conducted. This includes studies on hypoxia-immune signature^18^, cancer-associated alternative splicing event-related genes^19^, the miRNA-30 family^20–22^, and the KEAP1-NRF2-CUL3 axis^23^. Consequently, promising biomarker genes for the prognosis of HNSCC patients have been proposed. However, more efforts are needed to make better use of the TCGA-HNSCC database.

Autophagy is an advanced process of digesting the cytoplasm and organelles by autophagosomes and autolysosomes to protect cells, and thereby, cell components become an energy source by recycling^24,25^. Autophagy is also considered a strong promoter of metabolic homeostasis, as it has been shown to play an important role in the regulation of several survival and death signaling pathways that determine the cell fate of cancer^26–29^. On the other hand, antineoplastic agents, such as survivin inhibitors and disulfiram, may promote autophagic cell death in HNSCC cells, thus showing the opposite role of autophagy on cell survival^30,31^. The complex multi-step process of autophagy is tightly controlled by a set of autophagy-related genes (ARGs). Some ARGs have been shown to be associated with the prognosis of HNSCC patients using bioinformatics^29,32–34^. However, it has not been clarified how these ARGs exhibit their expression kinetics in an environment where autophagy occurs.

Serum starvation is the most widely studied method for inducing autophagy^35–38^. In HeLa cells, mitochondria-produced reactive oxygen species (ROS) are also known to induce autophagy via AMPK during starvation^39^. These recent advances in ARGs have prompted us to investigate whether genes containing ARGs that show altered expression profiles under serum starvation in vitro are associated with the prognosis of HNSCC patients. To determine this possibility, we investigated the effects of serum starvation on the biological activity of HNSCC cells under serum starvation and performed RNA sequencing of these cells. Then we extracted genes with large expression fluctuations in vitro and investigated the relationship between gene expression in tumors and normal solid tissues of TCGA-HNSCC patients and their prognosis. The results of this study suggest that, among the up-regulated genes under serum starvation, CALR, HSPA5, and TRIB3 are starvation-induced genes (SIGs) associated with the prognosis of TCGA-HNSCC patients.

## Materials and methods

### Cells

The human HNSCC cell lines SAS and Ca9-22 were obtained from the Japanese Collection of Research Bioresources (Tokyo, Japan). Cells were cultured in RPMI 1640 medium (Sigma-Aldrich, St. Louis, MO) supplemented with 10% fetal bovine serum (FBS) at 37 °C in a humidified atmosphere with 5% CO_2_.

### Cell proliferation assay and migration assay

For MTT assay, SAS cells were incubated with 3-(4,5-Dimethyl-2-thiazolyl)-2,5-diphenyl-2H-tetrazolium bromide reagent (DOJINDO, Osaka, Japan) for 2 h at 37 °C. At the end of each experiment, the medium was removed and 100 μL solution of 4% HCl 1 N in isopropanol was added to immediately dissolve the formazan crystals, and absorbance at 570 nm was recorded. For the migration assay, SAS cells were cultured in RPMI 1640 with 10% FBS until confluent. The cell layers were scratched using a plastic tip, as previously described^40^. The cells were further incubated in RPMI 1640 with/without FBS for 6 h. The closure rate of each scratched area was measured using ImageJ software, as previously described^40^.

### Transmission electron microscopy (TEM)

TEM was performed to observe SAS cells in serum-starved condition for 24 h. Serum-starved cells were washed with PBS, fixed in 2.5% glutaraldehyde in phosphate buffer, post-fixed in 2% osmium tetroxide, dehydrated in graded ethanol, and then embedded in epoxy resin. Ultrathin sections were stained with 2% uranyl acetate and observed using a JEM-1200 EX microscope (JEOL, Tokyo, Japan).

### RNA extraction

SAS cells were cultured in the absence of serum for 2 and 24 h. Total RNA from SAS cells were isolated using TRIsure (BIOLINE, Luckenwalde, Germany) according to the manufacturer’s instructions. We prepared two control samples, one 2 h sample, and one 24 h sample.

### RNA-sequencing and FASTQ file processing

According to the manufacturer's instructions, library preparation was performed using a TruSeq stranded mRNA sample prep kit (Illumina, San Diego, CA). Whole transcriptome sequencing was executed with the Illumina HiSeq 2500 platform in a 75-base single-end mode. Illumina Casava ver.1.8.2 software was used for base calling. Sequenced reads were mapped to the human reference genome sequences (hg19) using TopHat ver. 2.0.13 in combination with Bowtie2 ver. 2.2.3 and SAMtools ver. 0.1.19. Counts per gene were calculated with Cufflinks ver. 2.2.1. FPKMs and fragment counts were scaled via the median of the geometric means of fragment counts across all libraries.

### Analyzing the normalized counts data

We imported the normalized counts into Subio Platform v1.24.5849 (Subio Inc. Kagoshima, Japan)^41^ and all subsequent analyses were executed using this software. We set the lower limit as replacing positive numbers less than 10 with 10, and 0 counts with 8. Then, we calculated the log2 ratio against the geometric mean of the two control samples. We filtered out genes if their counts were always less than 15, or if their log2 ratios were between − 0.5 and 0.5 in all samples; a total of 6,363 genes remained after filtering. We extracted candidate DEGs by a twofold criterion.

### Analyzing TCGA-HNSC RNA-Seq data

We obtained and analyzed the RNA-Seq count data of TCGA-HNSC from the GDC Data Portal^42^ with the Subio platform. The workflow of TCGA RNA-Seq was the same as that applied to our RNA-seq data except for the thresholds. The lower limit for positive counts was 50, for 0 counts was 32, and the filter on counts was 50, and that on log2 ratios was between -1 and 1. In addition, the log ratios were taken against the average of solid normal tissue samples. For each of the 21 selected genes, we divided the primary tumor samples into two groups, those with count values higher or lower than the median, to compare the survival time with the Kaplan–Meier method.

### Pathways analysis and protein and protein interaction

The molecular pathways of the 21 selected genes were analyzed for gene ontology (GO) terms and Kyoto Encyclopedia of Genes and Genomes (KEGG) pathways using the Database for Annotation, Visualization, and Integrated Discovery (DAVID) server. GO enrichment was carried out over three primary levels: cellular components (CC), biological processes (BP), and molecular functions (MF). Based on the STRING online database (https://string-db.org/), we used these genes to establish a protein–protein interaction (PPI) network. Then, the most significant modules in the PPI networks were visualized.

### Statistical analyses

Statistical analyses were performed using the Student’s *t*-test with Microsoft Excel (Microsoft, Redmond, WA, USA). Results were expressed as the mean ± SD. Differences were considered significant at *P* < 0.05. For the survival analysis shown in Table 3, the hazard ratio (HR) relative to the indicated reference (ref) value, its 95% confidence interval (CI), and P-value (those of < 0.05 are indicated in bold) for the Cox hazard model are shown. The HR and its 95% CI were calculated by Cox regression analysis after proper evaluation of the assumptions of the Cox regression models with the use of the survival package.

## Results

### Effects of serum starvation on the biological activity of HNSCC cells

**Figure 1:**
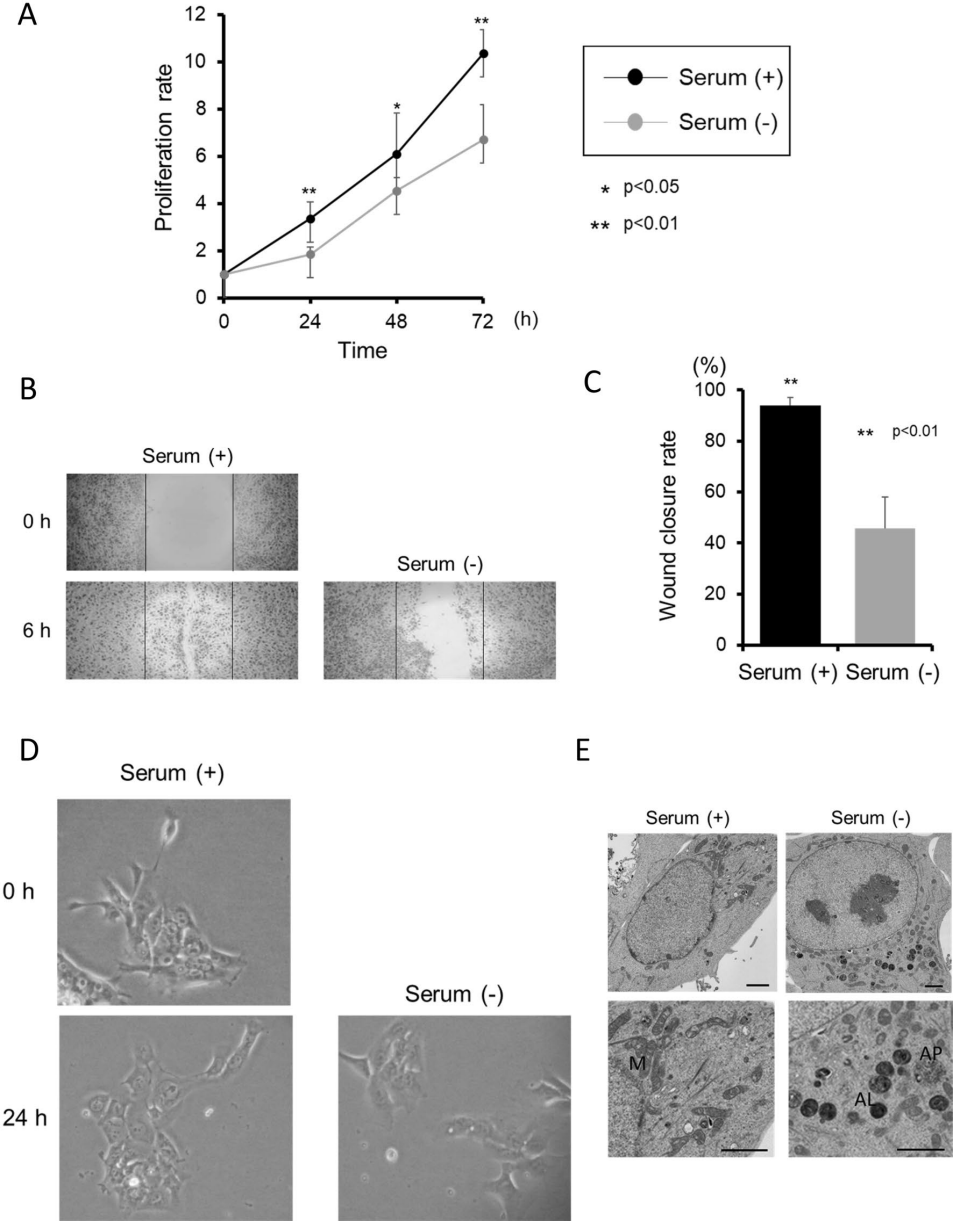
Effects of serum starvation on the biological activity of HNSCC SAS cells. (**A**) Growth of SAS cells as measured by MTT assay after treatment for the indicated time, with or without serum. The data are shown as mean ± SD of 3 independent experiments and analyzed by t-test. (**B**,**C**) Migration assay in cells treated with/without serum. The bars show the area of scratches at 0 h. The rates of wound closure were determined from the assays and indicate the mean ± SD of 3 independent experiments. (**D**) The cell morphology was photographed with a phase contrast microscope. (**E**) TEM examination of SAS incubated for 24 h with/without serum. The bar markers represent 2 μm. M: mitochondria, AP: autophagosome, AL: autolysosome.

We first examined the process of characterizing the response of HNSCC cells to serum starvation on cell proliferation, migration, and morphology. Following serum starvation, SAS cells proliferation and migration were considerably diminished, while cell morphology did not change (Fig. 1A–D). These findings were consistent with Ca9-22 cells proliferation, migration, and morphology mediated by serum starvation (Fig. S1A-D), and suggest that there is no much of differences between HNSCC cell lines. To determine morphological changes at the hyperfine structure level due to serum starvation, SAS cells were further investigated through TEM. Most SAS cells maintained in the presence of serum contained intact mitochondria that were distributed throughout the uniform cytoplasm. In cells cultured for 24 h in the absence of serum, autophagosomes and/or autolysosomes containing degraded mitochondria and dense structures, characteristic of autophagic cells not present in control cells, were observed (Fig. 1E). Based on the results in experiments on the biological activity of HNSCC cells under serum starvation, we decided to perform RNA- sequencing of SAS cells under serum starvation and extracted genes showing large fluctuations. We also investigated their expression in tumor tissues of TCGA-HNSCC patients and the relationship between their expression and the prognosis of patients. Fig. S2 shows the schedule of these experiments.

### RNA-sequencing of serum-starved SAS cells and altered expression of genes related to autophagy, cell growth, cell death, cell migration, cell proliferation, cell cycle, and cell adhesion

SAS cells were cultured in the absence of serum for 2 and 24 h and ARG expression was examined by RNA sequencing (Table 1 and Table S1), then we performed principal components analysis (PCA) and found that the expression profile did not change significantly after 2 h of starvation, but after 24 h of starvation, the expression profile changed (Fig. S3). At first, the altered expression of ARGs due to serum starvation was examined. To be consistent with PCA, a slight change in gene expression was observed 2 h after the onset of starvation. Some genes, such as DDIT3 and ERN1, were down-regulated after 2 h of starvation but increased after 24 h. After 24-h serum starvation, more than two-fold up-regulation was observed for 12 genes (ATP6V0A2, ATP6V1B1, ATP6V1C2, DDIT3, ERN1, NHLRC1, NUPR1, PIM2, TMEM150A, TRIB3, WIPI1, and XBP1) (Table 1). On the other hand, down-regulation of 50% or more was observed for 13 genes (BNIP3, BNIP3L, C10orf10, DAPK2, GAPDH, HMOX1, MEFV, PLK2, RRAGD, SESN3, SRPX, S100A8, and S100A9) (Table 1).

**Table 1:** Expression of autophagy-related genes under serum starvation.

ID	Description	NCBI gene ID	Fold change after 24 h	Fold change after 2 h
WIPI1	WD repeat domain, phosphoinositide interacting 1	55,062	2.0886614	0.930744261
XBP1	X-box binding protein 1	7494	2.1410127	0.878980954
NUPR1	Nuclear protein 1, transcriptional regulator	26,471	2.1501427	1.06686497
NHLRC1	NHL repeat containing E3 ubiquitin protein ligase 1	378,884	2.1578841	1.399708125
ATP6V0A2	ATPase H + transporting V0 subunit a2	23,545	2.3026843	1.142090599
ATP6V1B1	ATPase H + transporting V1 subunit B1	525	2.325067	0.828736747
PIM2	Pim-2 proto-oncogene, serine/threonine kinase	11,040	2.378625	1.229401772
TMEM150A	Transmembrane protein 150A	129,303	2.6394634	1.155387955
ATP6V1C2	ATPase H + transporting V1 subunit C2	245,973	2.6718903	1.774302383
ERN1	endoplasmic reticulum to nucleus signaling 1	2081	2.9943194	1.146213323
DDIT3	DNA damage-inducible transcript 3	1649	3.1741931	0.597946814
TRIB3	Tribbles pseudokinase 3	57,761	3.8404264	0.991618344
HMOX1	Heme oxygenase 1	3162	0.49524766	0.945472836
SRPX	Sushi repeat containing protein, X-linked	8406	0.4878078	0.857148158
RRAGD	Ras-related GTP-binding D	58,528	0.4833195	0.845809254
GAPDH	Glyceraldehyde-3-phosphate dehydrogenase	2597	0.4825641	0.94147729
C10orf10	Chromosome 10 open reading frame 10	11,067	0.46908534	1.273417327
PLK2	Polo-like kinase 2	10,769	0.40572378	0.835709592
BNIP3L	BCL2-interacting protein 3-like	665	0.37105525	0.898710392
SESN3	Sestrin 3	143,686	0.3578121	0.715624111
DAPK2	Death-associated protein kinase 2	23,604	0.34043416	0.718694176
S100A9	S100 calcium binding protein A9	6280	0.26209667	1.042621814
MEFV	MEFV, pyrin innate immunity regulator	4210	0.25515518	0.765465346
S100A8	S100 calcium binding protein A8	6279	0.24599823	1.095810293
BNIP3	BCL2-interacting protein 3	664	0.12660438	0.819784126

In the experiment using HNSCC cells in vitro, we confirmed suppressive effect of serum striation on cell proliferation and cell migration (Fig. 1), Therefore, RNA sequencing data obtained after starvation were further referred to as cell growth, cell death, cell migration, cell proliferation, cell cycle, and cell adhesion. Analyzed using 6 keywords (Table 2). After 24 h of serum starvation, more than two-fold up-regulation was observed for 425 genes. The top 5 genes were determined for each keyword. This included HSPA1A, OSGIN1, UCN, BST2, and SGK3 for cell growth, LOC728739, UCN, NPAS2, AGR2, and PYCR1 for cell death, BST2, ADGRA2, CALR, SGK3, and HSPA5 for cell migration, SGK3, IKZF3, SPTA1, MIR17HG, and CD22 for cell proliferation, ERN1, DDIT3, BEX2, CALR, and PIWIL4 for cell cycle, and AMIGO1, TNXB, TNC, FOXA2, and CD22 for cell adhesion (Table 2).

**Table 2:** Genes that were selected by 6 keywords and showed high up-regulation or down-regulation under serum starvation.

Key word	ID	Description	NCBI gene ID	Fold change after 24 h	Fold change after 2 h
Cell growth	HSPA1A	Heat shock protein family A (Hsp70) member 1A	3303	2.1397336	1.248177638
	OSGIN1	Oxidative stress-induced growth inhibitor 1	29,948	2.1668558	1.538782151
	UCN	Urocortin	7349	2.4790032	1.144155207
	BST2	Bone marrow stromal cell antigen 2	684	2.9329562	1.308922836
	SGK3	Serum/glucocorticoid regulated kinase family member 3	23,678	3.820176	1.176317287
	EDN1	Endothelin 1	1906	0.27418113	0.884857381
	PSRC1	Proline and serine rich coiled-coil 1	84,722	0.26818275	0.945457524
	S100A9	S100 calcium binding protein A9	6280	0.26209667	1.042621814
	S100A8	S100 calcium binding protein A8	6279	0.24599823	1.095810293
	CDKN2C	Cyclin-dependent kinase inhibitor 2C	1031	0.12124052	0.835693575
Cell death	LOC728739	Programmed cell death 2 pseudogene	728,739	2.3000002	1
	UCN	Urocortin	7349	2.4790032	1.144155207
	NPAS2	Neuronal PAS domain protein 2	4862	2.689144	0.930857474
	AGR2	Anterior gradient 2, protein disulphide isomerase family member	10,551	2.6983347	1.036070166
	PYCR1	Pyrroline-5-carboxylate reductase 1	5831	2.81048	1.165619558
	GPR37L1	G protein-coupled receptor 37 like 1	9283	0.3573708	0.929164088
	AKR1C3	Aldo–keto reductase family 1 member C3	8644	0.25129116	0.95490666
	CTSV	Cathepsin V	1515	0.24350072	0.872422377
	BNIP3	BCL2-interacting protein 3	664	0.12660438	0.819784126
	AXIN2	Axin 2	8313	0.07149426	0.979471303
Cell migration	BST2	Bone marrow stromal cell antigen 2	684	2.9329562	1.308922836
	ADGRA2	Adhesion G Protein-coupled receptor A2	25,960	3.3248994	1.09175771
	CALR	Calreticulin	811	3.6909635	0.82266943
	SGK3	serum/glucocorticoid regulated kinase family member 3	23,678	3.820176	1.176317287
	HSPA5	Heat shock protein family A (Hsp70) member 5	3309	7.4991713	0.83579081
	STC1	Stanniocalcin 1	6781	0.20706424	0.782881491
	ANLN	Anillin actin binding protein	54,443	0.18460144	0.933625648
	TCAF2	TRPM8 channel-associated factor 2	285,966	0.13127537	0.750317546
	ATOH8	atonal bHLH transcription factor 8	84,913	0.11830313	0.709818819
	SERPINB3	Serpin family B member 3	6317	0.11149136	1.039357318
Cell proliferation	SGK3	Serum/glucocorticoid regulated kinase family member 3	23,678	3.820176	1.176317287
	IKZF3	IKAROS family zinc finger 3	22,806	4.3976007	0.968962725
	SPTA1	Spectrin alpha, erythrocytic 1	6708	4.4961443	1.10674307
	MIR17HG	miR-17-92a-1 cluster host gene	407,975	4.602493	1.283155842
	CD22	CD22 molecule	933	5.679524	1.044954641
	ID2	Inhibitor of DNA binding 2	3398	0.17407766	1.119070975
	ATOH8	Atonal bHLH transcription factor 8	84,913	0.11830313	0.709818819
	SERPINB3	Serpin family B member 3	6317	0.11149136	1.039357318
	EGLN3	Egl-9 family hypoxia inducible factor 3	112,399	0.100901835	0.74969137
	AXIN2	Axin 2	8313	0.07149426	0.979471303
Cell cycle	ERN1	Endoplasmic reticulum to nucleus signaling 1	2081	2.9943194	1.146213323
	DDIT3	DNA damage-inducible transcript 3	1649	3.1741931	0.597946814
	BEX2	Brain expressed X-linked 2	84,707	3.5948327	1.06210961
	CALR	Calreticulin	811	3.6909635	0.82266943
	PIWIL4	Piwi like RNA-mediated gene silencing 4	143,689	3.979958	1.182165243
	CENPE	Centromere protein E	1062	0.2240672	0.891558939
	KCTD11	Potassium channel tetramerization domain containing 11	147,040	0.20453872	0.83617986
	MAP2K6	Mitogen-activated protein kinase kinase 6	5608	0.19298029	0.761199796
	ID2	Inhibitor of DNA binding 2	3398	0.17407766	1.119070975
	CDKN2C	Cyclin-dependent kinase inhibitor 2C	1031	0.12124052	0.835693575
Cell adhesion	AMIGO1	Adhesion molecule with Ig like domain 1	57,463	2.892857	1.089285831
	TNXB	Tenascin XB	7148	3.0829856	1.187939406
	TNC	Tenascin C	3371	3.1931474	1.035432055
	FOXA2	Forkhead box A2	3170	3.3726099	0.838221697
	CD22	CD22 molecule	933	5.679524	1.044954641
	CXCL8	C-X-C motif chemokine ligand 8	3576	0.3170054	0.587415932
	SERPINI1	Serpin family I member 1	5274	0.31145853	0.953841739
	CDH2	Cadherin 2	1000	0.29494244	1.09390035
	CNTN1	Contactin 1	1272	0.26283494	0.835311109
	CCL2	C–C motif chemokine ligand 2	6347	0.22852589	0.886680707

After 24 h of serum starvation, over 50% down-regulation was observed for 733 genes. The top 5 genes with the largest reductions were: EDN1, PSRC1, S100A9, S100A8, and CDKN2C for cell growth, GPR37L1, AKR1C3, CTSV, BNIP3, and AXIN2 for cell death, STC1, ANLN, TCAF2, ATOH8, and SERPINB3 for cell migration, ID2, ATOH8, SERPINB3, EGLN3, and AXIN2 for cell proliferation, CENPE, KCTD11, MAP2K6, ID2, and CDKN2C for cell cycle, and CXCL8, SERPINI1, CDH2, CNTN1, and CCL2 for cell adhesion (Table 2).

### Expression of serum starvation-induced genes in TCGA-HNSCC patients

**Figure 2:**
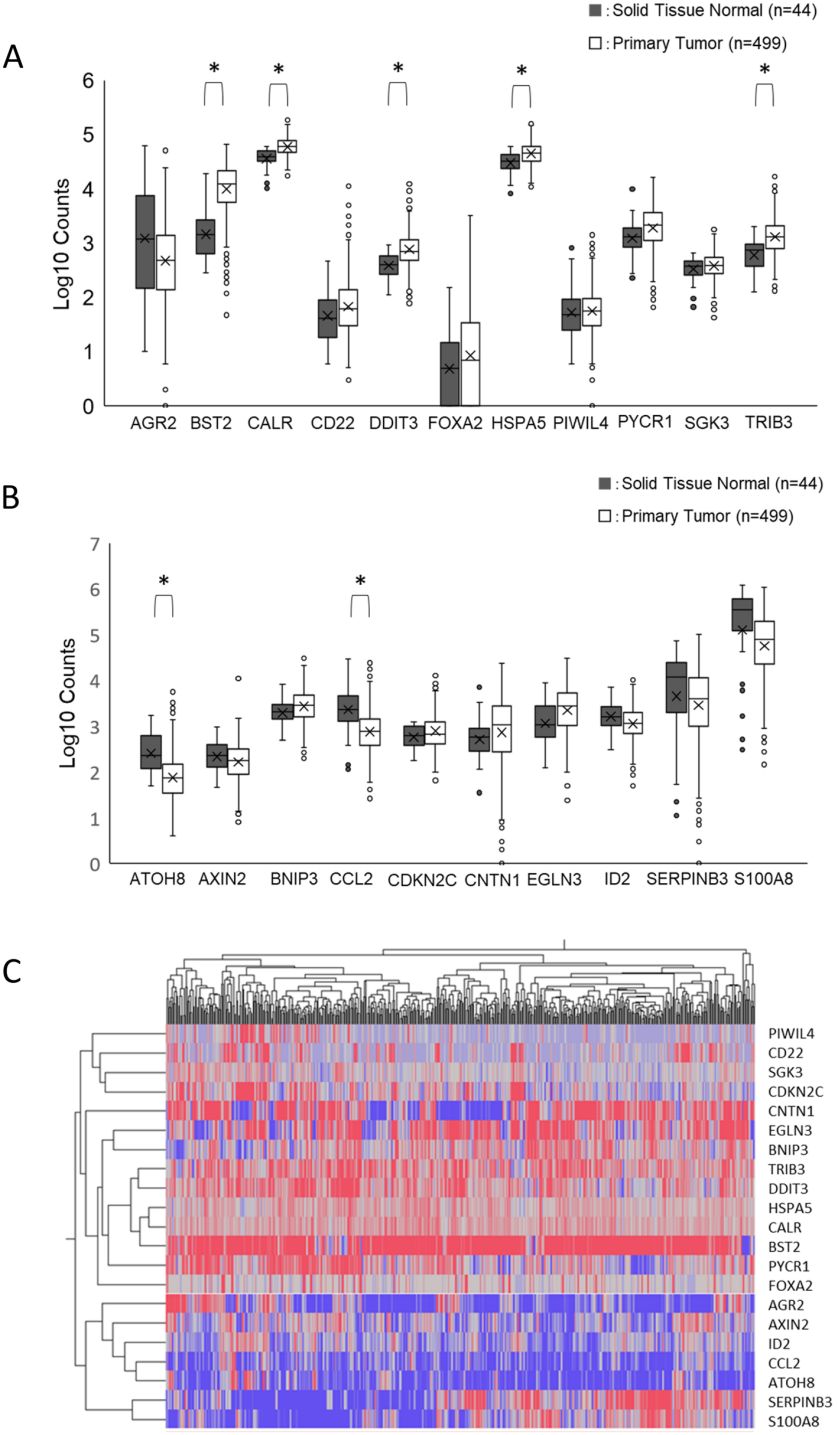
Expression of serum starvation-induced genes (SIGs) in TCGA-HNSCC patients. The expression of 21 genes that showed significant changes in expression by serum starvation and their relative expression levels in TCGA-HNSCC patients were determined in primary tumors and solid normal tissues. (**A**) Box plots of the expression of 11 genes (AGR2, BST2, CALR, CD22, DDIT3, FOXA2, HSPA5, PIWIL4, PYCR1, SGK3, and TRIB3) that have been up-regulated more than two-fold. (**B**) Box plots of the expression of 10 genes (ATOH8, AXIN2, BNIP3, CCL2, CDKN2C, CNTN1, EGLN3, ID2, SERPINB3, and S100A8) that were down-regulated by more than 50%. * P < 0.05. (**C**) Heat map of 21 SIG expression profiles. Colors from blue to red indicate low to high expression levels.

From the 70 genes altered in HNSCC cells by 24 h serum deficiency, the top two genes showing significant expression changes were selected for each of the 7 keywords, including autophagy, cell proliferation, cell death, cell migration, cell proliferation, cell cycle, and cell adhesion. Of the 28 genes selected, 6 were associated with replication. Moreover, the microRNA MIR17HG was excluded. Therefore, we finally focused on 21 genes. Of these, 11 were up-regulated genes and 10 were down-regulated genes. When the expression of these SIGs was examined in TCGA-HNSCC patients, 9 of the 11 up-regulated genes were also up-regulated in the primary tumor compared to solid normal tissue. Significant expression differences were observed in BST2, CALR, DDIT3, HSPA5, and TRIB3 (Fig. 2A). On the other hand, 6 out of the 10 down-regulated genes had reduced expression in tumors compared to solid normal tissue, with significant differences observed in the ATOH8 and CCL2 genes (Fig. 2B). A heat map was also created to represent the level of up-or down-regulated expression profiles of 21 genes (Fig. 2C).

### Function and PPI analysis of SIGs

**Figure 3:**
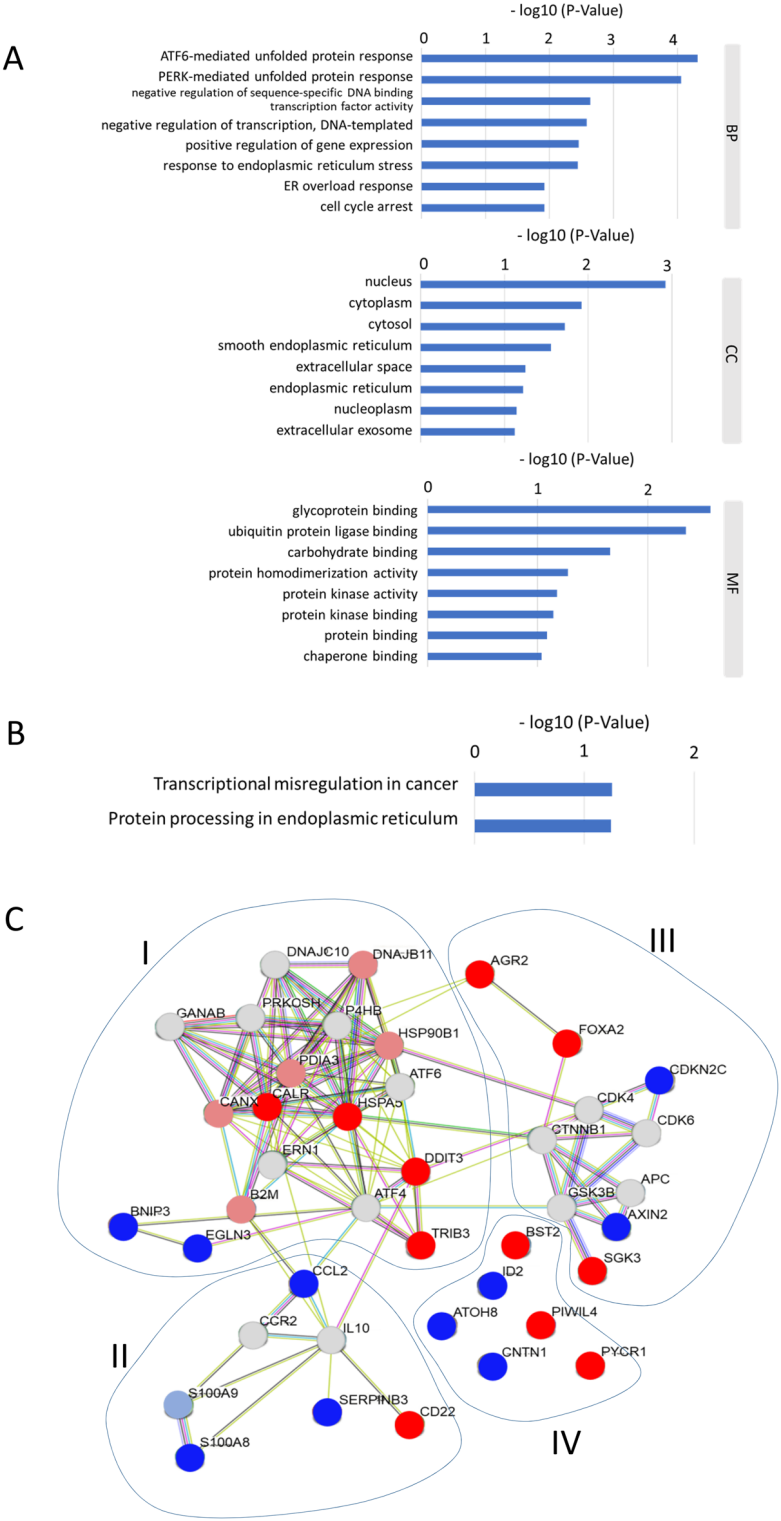
Function and protein–protein interaction analysis of SIGs. (**A**) A list of the top 8 significant GO terms determined by GO enrichment analysis of 21 selected genes. BP, biological process; CC, cellular composition; MF, molecular function. (**B**) List of molecular pathways determined by KEGG pathway enrichment analysis of 21 selected genes. (**C**) Proteins encoded by 21 genes extracted using 7 keywords were subjected to PPI network analysis. Up-regulated genes are shown in red. Down-regulation is shown in blue, and gray indicates genes whose expression did not change under serum starvation.

GO and KEGG enrichment pathway analyses were performed to investigate the biological properties and potential signaling pathways of the 21 selected genes. Using GO enrichment analysis, enriched terms were ATF6-mediated unfolded protein response, PERK-mediated unfolded protein response, negative regulation of sequence-specific DNA-binding transcription factor activity, and negative regulation of transcription. These GO terms were associated with several important biological processes including DNA-templated gene expression response to endoplasmic reticulum (ER) stress, ER stress response, and positive regulation of cell cycle arrest (Fig. 3A). KEGG analysis showed that the prognostic genes were significantly enriched in pathways of transcriptional misregulation in cancer and protein processing in the endoplasmic reticulum (Fig. 3B). In PPI network analysis, 21 genes were subdivided into 4 clusters (I-IV). In cluster I, up-regulated genes, CALR, HSPA5, DDIT3, and TRIB3, formed a close interaction network (Fig. 3C). PIWIL4 and PYCR1 in cluster IV were not associated with other up-regulated genes.

### Prognostic significance of 21 SIGs in TCGA-HNSCC patients

**Figure 4:**
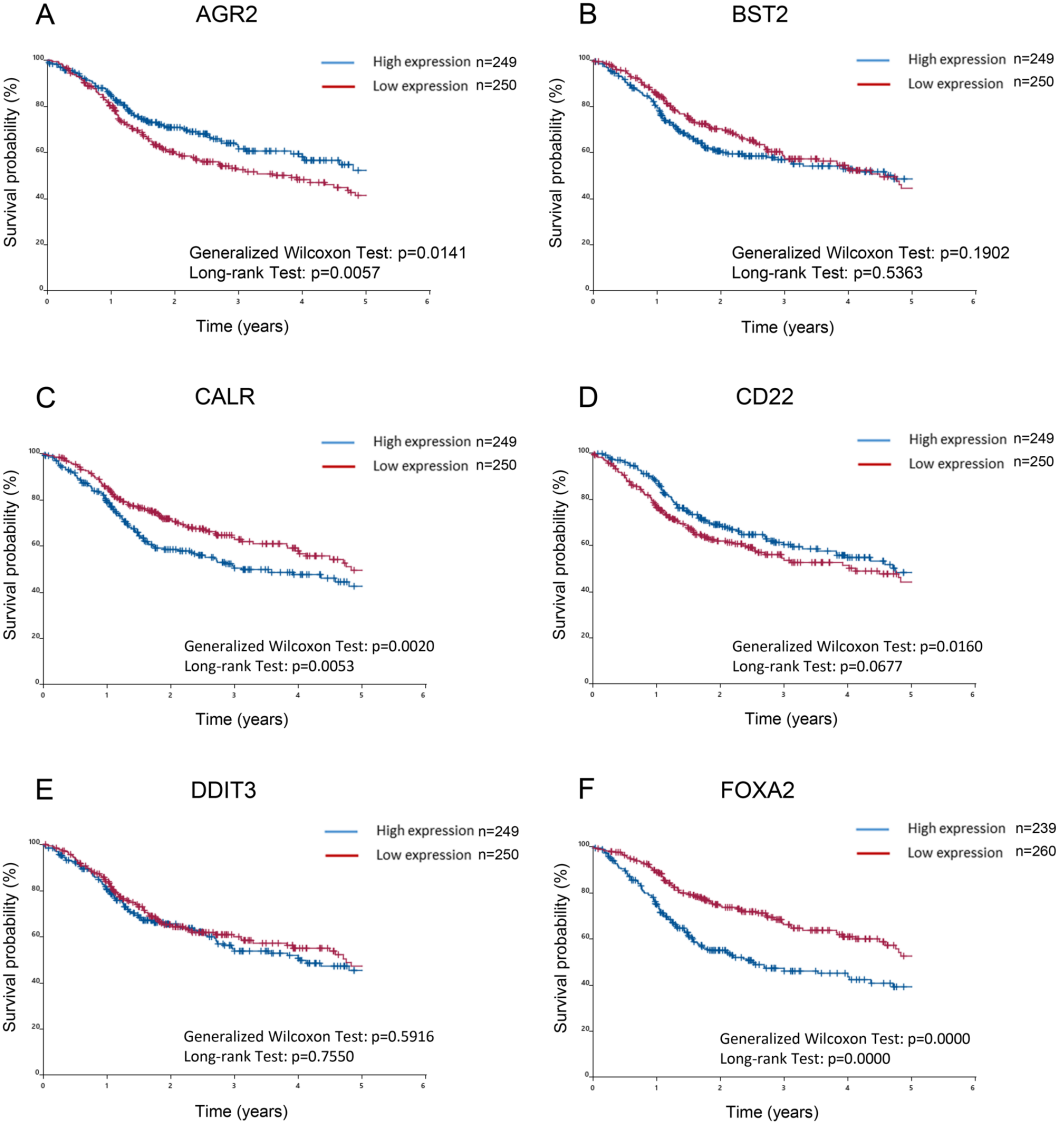

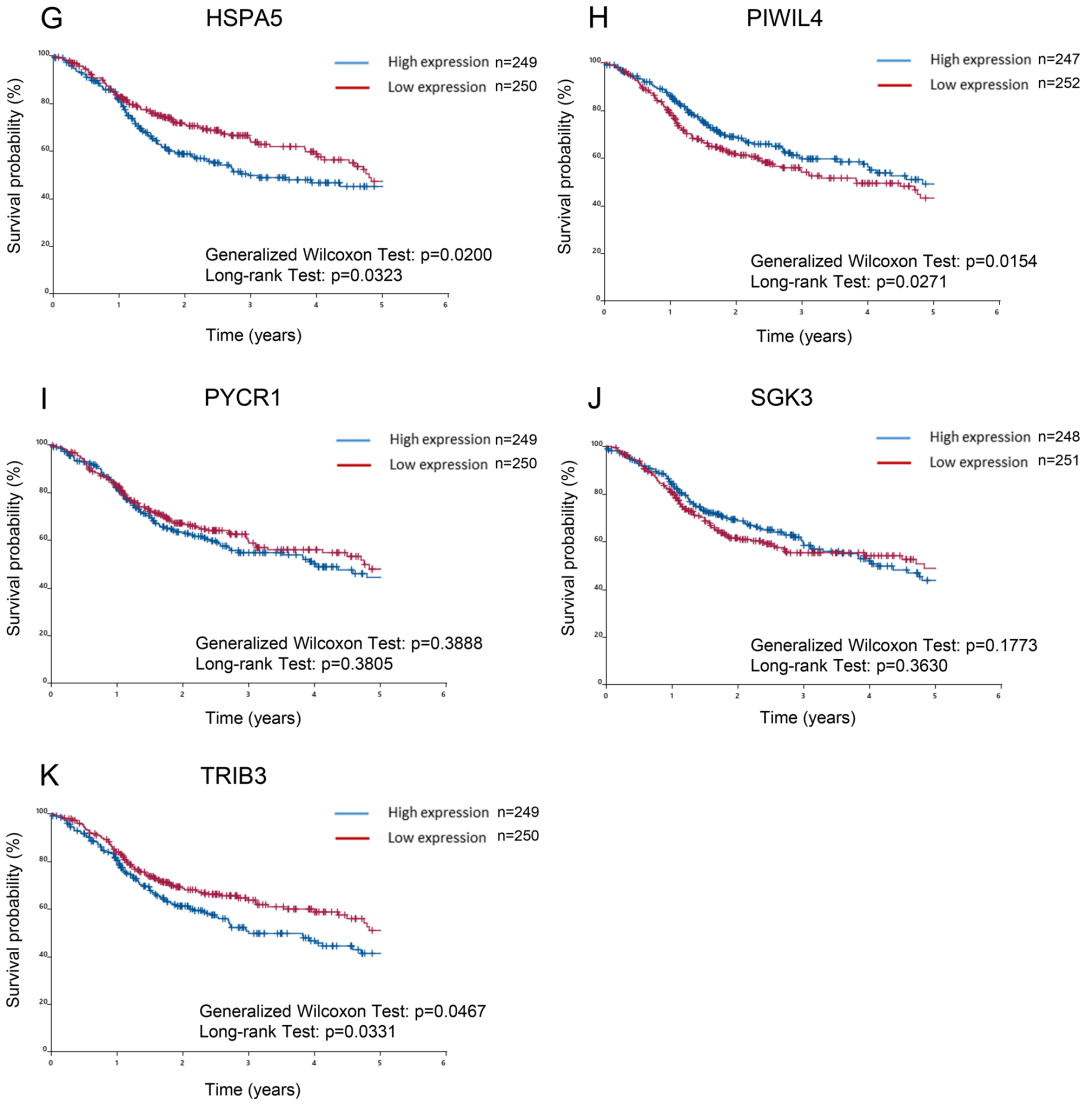
Prognostic significance of SIGs in TCGA-HNSCC patients. Overall survival of TCGA-HNSCC patients, classified by 11 up-regulated SIG expression levels, was determined by the Kaplan–Meier method. The difference in survival time determined by the Kaplan–Meier method was examined using the generalized Wilcoxon test and the long rank test. (**A**) AGR2, (**B**) BST2, (**C**) CALR, (**D**) CD22, (**E**) DDIT3, (**F**) FOXA2, (**G**) HSPA5, (**H**) PIWIL4, (**I**) PYCR1, (**J**) SGK3, (**K**) TRIB3.

We investigated whether SIGs that the differentially expressed SIGs in tumors and normal tissues of TCGA-HNSCC patients was associated with prognosis. Patients were divided into two groups based on the expression of SIGs. The expression levels of patients in the high expression group were higher than the median, and the remaining patients were classified in the low expression group^43,44^. The difference in survival time determined by the Kaplan–Meier method was examined using the generalized Wilcoxon test and the long rank test (Fig. 4). Among the up-regulated SIGs, high expression of CALR (Fig. 4C), FOXA2 (Fig. 4F), HSPA5 (Fig. 4G), and TRIB3 (Fig. 4K) was correlated with decreased patient survival. FOXA2 was excluded in subsequent studies because it was not significantly up-regulated in tumors compared to normal solid tissue. Conversely, high expression at AGR2 (Fig. 4A) and PIWIL4 (Fig. 4H) were correlated with significant improvement in overall patient survival. On the other hand, high expression of BST2 (Fig. 4B), CD22 (Fig. 4D), DDIT3 (Fig. 4E), PYCR1 (Fig. 4I), and SGK3 (Fig. 4J) was not associated with patient survival. The Kaplan–Meier method was also applied to down-regulated genes, but there was no association between gene expression and patient survival in TCGA-HNSCC patients (Fig. S4).

**Figure 5:**
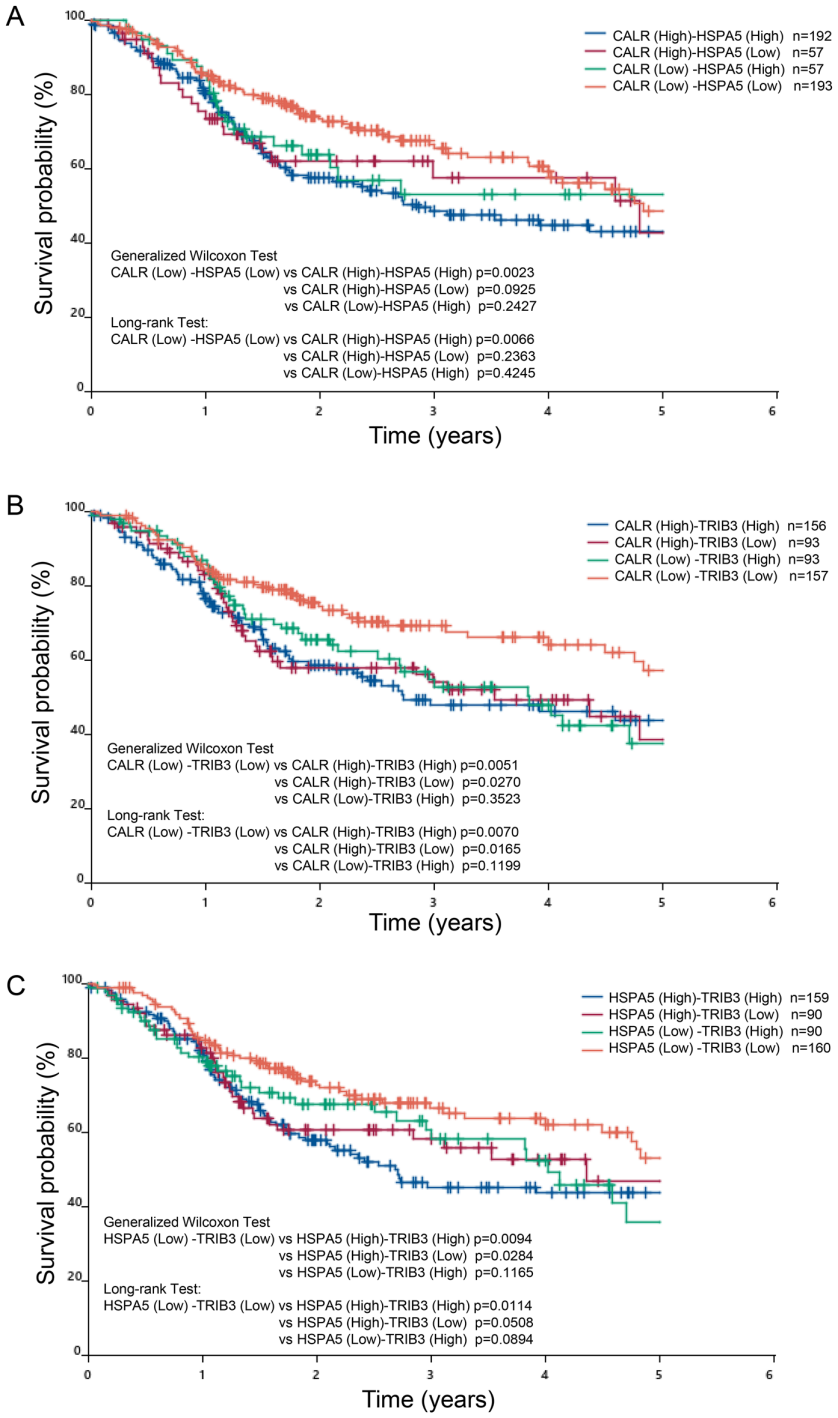
Prognostic significance of combinational expression of CALR, HSPA5, and TRIB3 in TCGA-HNSCC patients. The survival curve was recalculated based on the expression of CALR, HSPA5, and TRIB3. (**A**) Survival curves for high CALR- high HSPA5 group, high CALR-low HSPA5 group, low CALR-high HSPA5 group, and low CALR-low HSPA5 group. (**B**) Survival curves for high CALR-high TRIB3 group, high CALR-low TRIB3 group, low CALR-high TRIB3 group, and low CALR-low TRIB3 group. (**C**) Survival curves for high HSPA5-high TRIB3 group, high HSPA5-low TRIB3 group, low HSPA5-high TRIB3 group, and low HSPA5-low TRIB3 group.

When the survival curve was recalculated based on the expression of CALR, HSPA5, and TRIB3, the probability of survival in the high- and high group combinations was much lower than in the low- and low group combinations, predicting patient prognosis. It shows the high ability of group combination to do (Fig. 5).

### Cox regression analysis of the association of SGIs and classical prognostic factors with survival in the TCGA-HNSCC patients

Expression of CALR, HSPA5, and TRIB3 was correlated with reduced overall survival in patients with TCGA-HNSCC, so these genes were further analyzed. Univariate and multivariate analysis (Cox proportional hazard model) was performed using the three genes and classical risk factors, such as gender, HPV, smoking, age, and TNM stage, as independent variables. In univariate analysis, CALR-High (vs. Low) (HR = 1.416, 95% CI = 1.069–1.875, p = 0.015), HSPA5-High (vs. Low) (HR = 1.362, 95% CI = 1.029–1.804, p = 0.031), TRIB3-High (vs Low) (HR = 1.361, 95% CI = 1.028–1.803, p = 0.031), age (HR = 1.016, 95% CI = 1.003–1.029, p = 0.015), sex (HR = 0.721, 95% CI = 0.535–0.973, p = 0.032), M stage (HR = 4.748, 95% CI = 1.749–12.889, p = 0.002), and N stage (HR = 1.078, 95% CI = 1.002–1.160, p = 0.045) were significantly correlated with the prognosis of TCGA-HNSCC patients (Table 3). Multivariate analysis showed that the combination of two genes (CALR-High and HSPA5-High) (P = 0.022) and three genes (P = 0.027) did not make a clear difference in correlation.

**Table 3:** Univariate and multivariate analyses of three genes (CALR, HSPA5, and TRIB3) using the TCGA-HNSC patient data.

	Univariate			Multivariate		
	HR	95%CI	P-value	HR	95%CI	P-value
CALR_High(vs.Low)	1.416	1.069–1.875	**0.015**	–		
HSPA5_High(vs.Low)	1.362	1.029–1.804	**0.031**	–		
TRIB3_High(vs.Low)	1.361	1.028–1.803	**0.031**	–		
**Gene(CALR-HSPA5-TRIB3)**						
Low–Low–Low	1	ref		1	ref	
High–Low–Low	1.208	0.607–2.405	0.590	1.455	0.696–3.043	0.319
Low–High–Low	1.326	0.805–2.186	0.268	1.398	0.833–2.348	0.205
Low–Low–High	0.733	0.290–1.850	0.510	0.691	0.245–1.949	0.485
High–High–Low	1.783	1.118–2.845	**0.015**	1.777	1.086–2.907	**0.022**
High–Low–High	1.901	1.002–3.608	**0.049**	1.805	0.934–3.488	0.079
Low–High–High	1.861	1.061–3.265	**0.030**	1.887	1.021–3.486	**0.043**
High–High–High	1.619	1.088–2.410	**0.018**	1.614	1.056–2.467	**0.027**
Age(per1year)	1.016	1.003–1.029	**0.015**	1.023	1.009–1.038	**0.002**
Sex_male(vs.female)	0.721	0.535–0.973	**0.032**	0.862	0.611–1.215	0.396
HPVstatus_Positive(vs.Negative)	0.777	0.524–1.153	0.210	0.784	0.505–1.218	0.279
Alcohol_history_Yes(vs.No)	0.947	0.701–1.279	0.723	1.051	0.75–1.472	0.774
Cigarettesperday_>0(vs.0)	0.960	0.724–1.272	0.774	0.963	0.709–1.309	0.811
Mstage_m1(vs.m0)	4.748	1.749–12.889	**0.002**	4.979	1.699–14.592	**0.003**
Nstage(Continuousvariableper1)	1.078	1.002–1.160	**0.045**	1.112	1.023–1.208	**0.013**
**Nstage (category)**						
n0	1	ref				
n1	1.061	0.713–1.581	0.769			
n2	1.679	0.898–3.138	0.105			
n2a	1.607	0.744–3.469	0.227			
n2b	0.977	0.632–1.510	0.916			
n2c	1.980	1.229–3.190	**0.005**			
n3	1.698	0.623–4.624	0.300			
Tstage(Continuousvariableper1)	0.999	0.902–1.106	0.986	0.979	0.877–1.092	0.700
**Tstage (category)**						
t1	1	ref				
t2	1.092	0.57–2.092	0.790			
t3	1.421	0.746–2.705	0.285			
t4	1.543	0.681–3.496	0.299			
t4a	1.056	0.554–2.015	0.868			
t4b	1.984	0.439–8.960	0.373			

HR: hazard ratio; 95% CI: 95% confidence interval; ref: reference value.

## Discussion

Autophagy has been suggested to be a biological marker for estimating the prognosis of cancer patients. In a previous HNSCC bioinformatics study, Li et al.^29^ identified a novel autophagy-related signature consisting of three hub genes, MAP1LC3B, FADD, and LAMP1, that may provide promising biomarker genes for the treatment and prognosis of HNSCC. Similarly, Jin et al.^33^ determined 35 genes for HNSCC and identified ITGA3, CDKN2A, FADD, NKX2-3, BAK1, CXCR4, and HSPB8 as prognostic ARGs. Ren et al.^34^ also reported 13 ARGs as genes that predict prognosis. In the present study, HNSCC cells were cultured under serum starvation, which can efficiently induce autophagy, and RNA sequencing was used to examine the expression of ARGs.

FBS is commonly used as a supplement to animal cell culture medium^45^. Additionally, FBS consists of several compositions such as macromolecules, carrier proteins for lipoid substances and trace elements, attachment and spreading factors, low molecular weight nutrients, hormones, and growth factors^45^. Among them, growth factors were reported to influence cell proliferation, migration, survival, and morphogenesis^46^. Under serum starvation, SAS cells, a high-risk HPV-negative HNSCC cell line^47^, showed no significant changes in cell morphology after 24 h, but cell growth and migration capacity were suppressed. Serum starvation showed no significant effect on deforming cell morphology under microscopy. However, electron micrographs revealed the presence of autophagosomes and mitochondrial phagocytosis, being consistent with the features during autophagy of SAS cells^27^. This suggested that autophagy was induced in this serum-deficient situation. After 24-h starvation, mRNA sequencing of SAS cells detected 12 up-regulated ARGs (ATP6V0A2, ATP6V1B1, ATP6V1C2, DDIT3, ERN1, NHLRC1, NUPR1, PIM2, TMEM150A, TRIB3, WIPI1, and XBP1) and 13 down-regulated ARGs (BNIP3, BNIP3L, C10orf10, DAPK2, GAPDH, HMOX1, MEFV, PLK2, RRAGD, SESN3, SRPX, S100A8, and S100A9), again supporting the induction of autophagy of SAS cells under serum starvation. These genes differed from the ARGs previously reported to predict the prognosis of HNSCC patients^29,33,34^. This starvation-induced approach may be beneficial in extrapolating ARGs that have not been previously identified as differentially expressed genes. In addition, as with ARGs, we also found aberrant expression of genes related to cell growth, cell death, cell migration, cell proliferation, cell cycle, and cell migration (Fig. S2). Finally, 21 SIGs that showed significant up-regulation or down-regulation were selected. Comparing how these genes were expressed in normal and cancer tissues in TCGA-HNSCC patients, we found 11 genes that were more strongly expressed in cancer cells and 10 genes that were down-regulated in cancer tissues. Among them, BST2, CALR, DDIT3, HSPA5, and TRIB3 were significantly up-regulated in cancer tissues.

GO and KEGG analyses revealed the involvement of ATF6-mediated unfolded protein responses and PERK-mediated unfolded protein responses mainly in the nucleus, and the ability of SIGs to bind glycoproteins and ubiquitin protein ligases. In addition, networking between CALR, HSPA5, DDIT3, and TRIB3 was demonstrated by PPI analysis as a cluster. Consistent with the PPI analysis results, when TCGA-HNSCC patients were divided into high-expression and low-expression groups, and then analyzed by the Kaplan–Meier method, CALR, FOXA2, HSPA5, and TRIB3 were found to be correlated with reduced survival. FOXA2 was excluded because its expression was not significantly increased in tumors compared to normal tissues in TCGA-HNSCC patients. In contrast, some in vitro up-regulated SIGs, such as AGR2 and CD22, showed no significant difference, but survival was inversely proportional to that of CALR, FOXA2, HSPA5, and TRIB3*.* This may be due to the fact that there was no significant difference in AGR2 and CD22 expression between tumors and normal tissues (Fig. 2A,B). On the other hand, if there is a clear difference in survival, high expression of these genes may be applicable to predict a better prognosis for patients. Recalculation of the survival curve between CALR, HSPA5, and TRIB3 showed that comparing the combination of the two high groups with te combination of the low groups significantly reduced the probability of survival (Fig. 5). Furthermore, cox regression analysis confirmed that three SIGs (CALR, HSPA5, and TRIB3), sex, M-stage, and N-stage were associated with survival in HNSCC patients. This suggests that CALR, HSPA5, and TRIB3 are predictors of poor prognosis. Since the combination of two genes (CALR-High and HSPA5-High) and three genes did not make a clear difference in correlation (Table 3), patients will have a poor prognosis, especially when both CALR and HSPA5 are highly expressed.

Calreticulin, CALR, is a soluble multifunctional protein found in the ER lumen and is involved in calcium homeostasis, transcriptional regulation, immune response, and cellular function^48,49^. It is expressed at higher levels in many cancerous tissues than in normal tissues. High CALR expression is correlated with both advanced clinical stage and lymph node metastasis^50–52^. CALR has been shown to promote cell motility and enhance resistance to anoikis through STAT3-CTTN-AKT pathway of esophageal SCC^53^. Positive CALR staining was observed in the majority of tumor case (96%) of the oral cavity, whereas the incidence was lower in non-cancerous matching tissue cases (32%). It was also been reported that stable knockdown of CALR in oral cancer cells reduced cell proliferation^50^. The unfolding protein response (UPR) is a cellular stress response related with ER stress. One of the proteins involved in this UPR is Heat shock 70 kDa protein 5/glucose-regulated protein (HSPA5/GRP78). HSPA5 is the master regulator of UPR and is associated with tumor progression, tumor size, and poor prognosis^54–57^. In situations where protein production is required for tumor growth, USPA5 is overactivated to process a high flux of protein passing through the ER, maintaining ER homeostasis. Expression of HSPA5 is induced by glucose starvation^58,59^. Correspondingly, HSPA5 has been reported to be up-regulated in tumors of various organs such as breast, liver, stomach, esophagus, brain, prostate, head and neck, and melanoma, and may be accompanied by aggressive tumor behavior and recurrence^60,61^. A comprehensive proteomic analysis of oral SCCs also showed up-regulation of three members of the HSP family, including HSP90, HSPA5 and HSPA8^62^.

Tribbles homologue 3, TRIB3, is a member of the mammalian pseudokinase tribble family and is involved in multiple biological processes including the cellular response to glucose deficiency stress and ER stress. Several studies have shown that TRIB3 is elevated in multiple cancer cell lines and primary tumors including colorectal cancer, breast cancer, and lung cancer. In renal cancer, TRIB3 is overexpressed compared to normal tissue and is associated with tumor progression and poor prognosis^63–65^. In the tongue SCC, both TRIB3 and AKT were highly expressed compared to adjacent non-cancerous tissues, correlating TRIB3 overexpression with tumor pathological T stage, lymph node metastasis, and tumor recurrence. However, when TRIB3 was overexpressed in tongue SCC cells using a viral vector, phosphorylated AKT protein was reduced^66^. All of these genes, CALR, HSPA5, and TRIB3, are associated with ER stress. Their up-regulation may be a promising biomarker for predicting the prognosis of HNSCC.

There are several past studies where in vitro events and RNA-sequencing data were linked to informatics analysis of HNSCC patients^67,68^. You et al.^67^ established radiation-resistant cells by repeated irradiation in vitro and identified radioresistant genes using the TCGA-HNSCC database. In the present study, by analyzing genes induced by serum starvation of HNSCC cells, we detected genes that could not be obtained by previous TCGA database analysis and show their usefulness in predicting the prognosis of HNSCC patients. This approach may help to understand the genetic response of cancer cells to ER stress under therapeutic processes such as radiation therapy and chemotherapy.

## Conclusions

Up-regulated and down-regulated genes associated with serum starvation using HNSCC cells were identified. Expression of HSPA5, TRIB3, and CALR in SAS cells was up-regulated by in vitro serum starvation and up-regulated in TCGA-HNSCC tissue tumors. High expression of these genes was closely associated with reduced survival in patients with TCGA-HNSCC. These SIGs have the potential to be molecular prognostic markers in HNSCC patients.

## Supplementary Information

Supplementary Information 1.

Supplementary Information 2.

## Abbreviations

SCC: Squamous cell carcinoma
HNSCC: Head and neck squamous cell carcinoma
TCGA: The Cancer Genome Atlas
CALR: Calreticulin
HSPA5: Heat shock protein family A (Hsp70) member 5
TRIB3: Tribbles pseudokinase 3
