# Supplementary material for: Prognostic association of starvation-induced gene expression in head and neck cancer
Source: Sci Rep. 2021 Sep 27;11:19130. doi: 10.1038/s41598-021-98544-1 (PMC8476550; doi:10.1038/s41598-021-98544-1)
Supplement: Supplementary file 1 — Supplementary Information 1. [file 41598_2021_98544_MOESM1_ESM.pptx]

## Slide 1
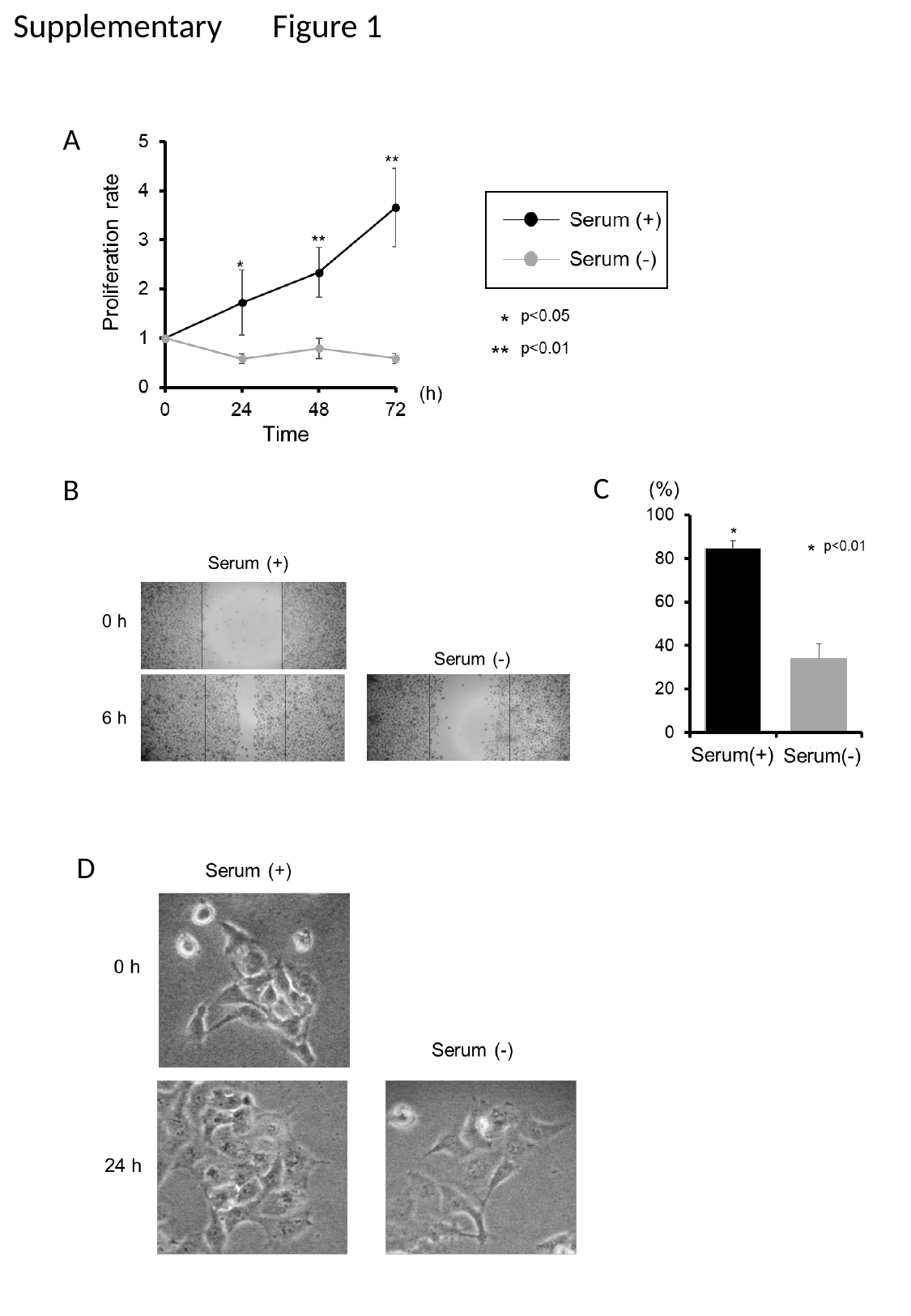

Supplementary　Figure 1
A
C
B
D

## Slide 2
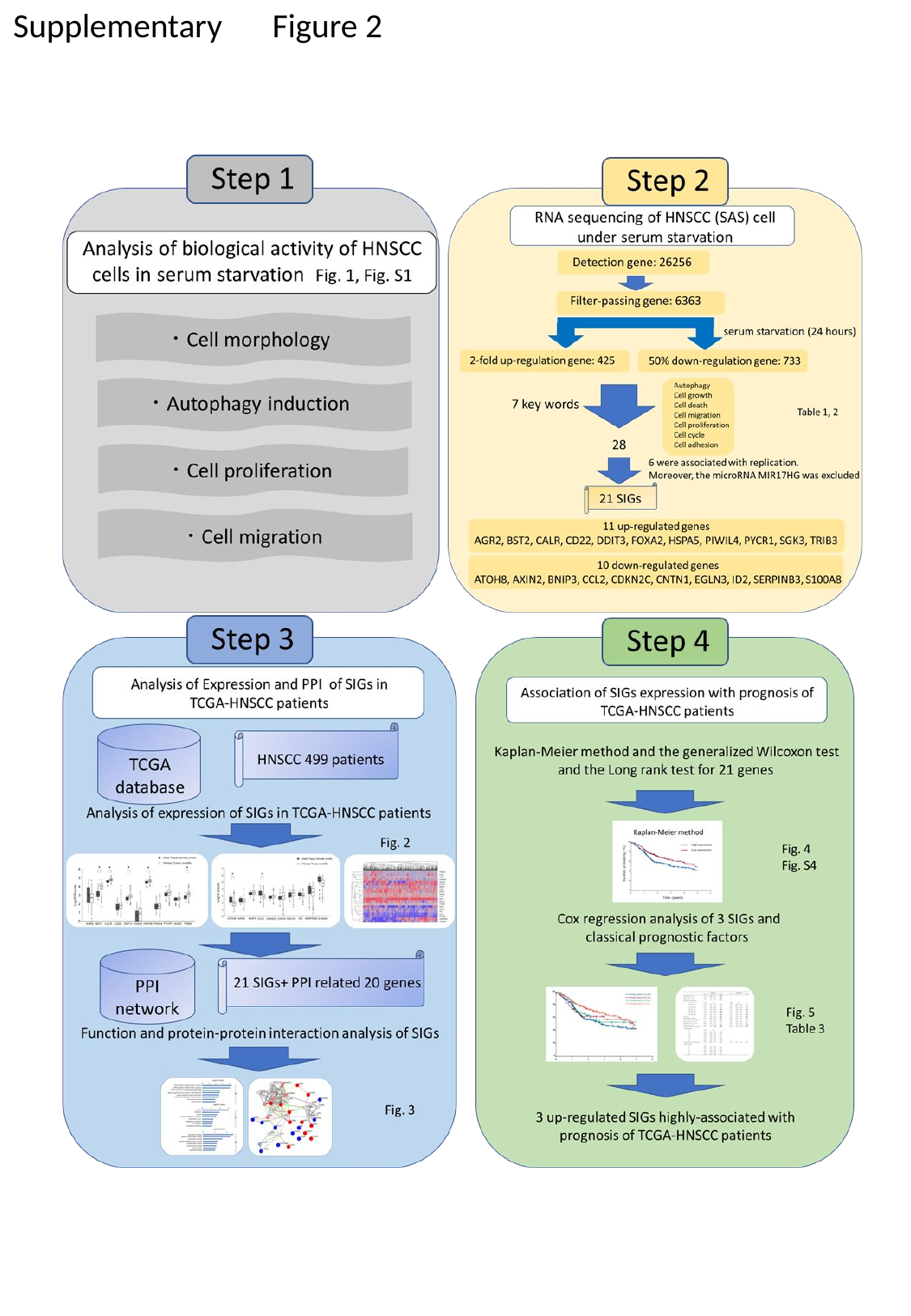

Supplementary　Figure 2

## Slide 3
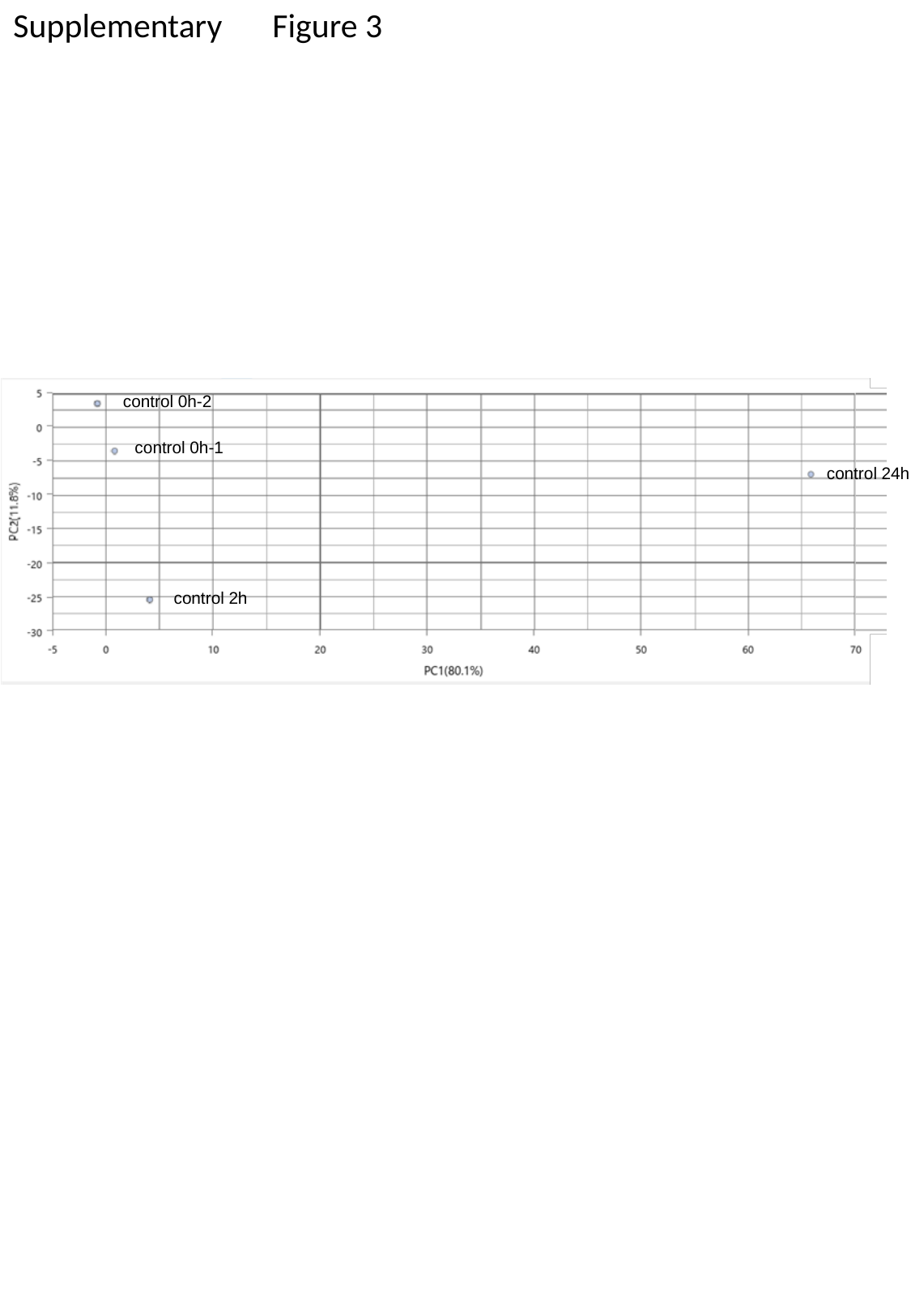

Supplementary　Figure 3
control 0h-2
control 0h-1
control 24h
control 2h

## Slide 4
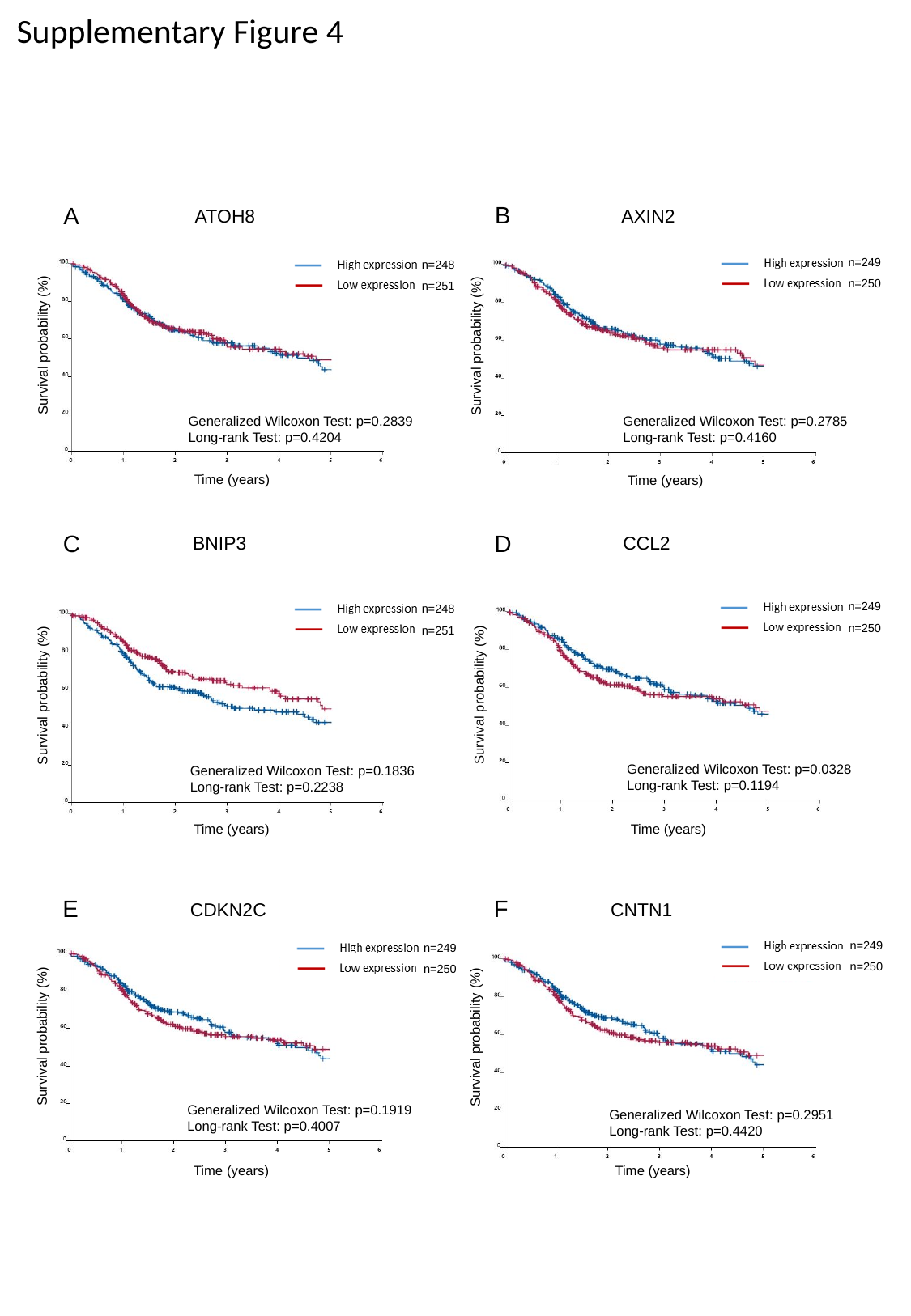

Supplementary Figure 4
B
A
ATOH8
AXIN2
n=249
n=250
n=248
n=251
Survival probability (%)
Survival probability (%)
Generalized Wilcoxon Test: p=0.2839
Long-rank Test: p=0.4204
Generalized Wilcoxon Test: p=0.2785
Long-rank Test: p=0.4160
Time (years)
Time (years)
D
C
BNIP3
CCL2
n=249
n=250
n=248
n=251
Survival probability (%)
Survival probability (%)
Generalized Wilcoxon Test: p=0.0328
Long-rank Test: p=0.1194
Generalized Wilcoxon Test: p=0.1836
Long-rank Test: p=0.2238
Time (years)
Time (years)
F
E
CDKN2C
CNTN1
n=249
n=250
n=249
n=250
Survival probability (%)
Survival probability (%)
Generalized Wilcoxon Test: p=0.1919
Long-rank Test: p=0.4007
Generalized Wilcoxon Test: p=0.2951
Long-rank Test: p=0.4420
Time (years)
Time (years)

## Slide 5
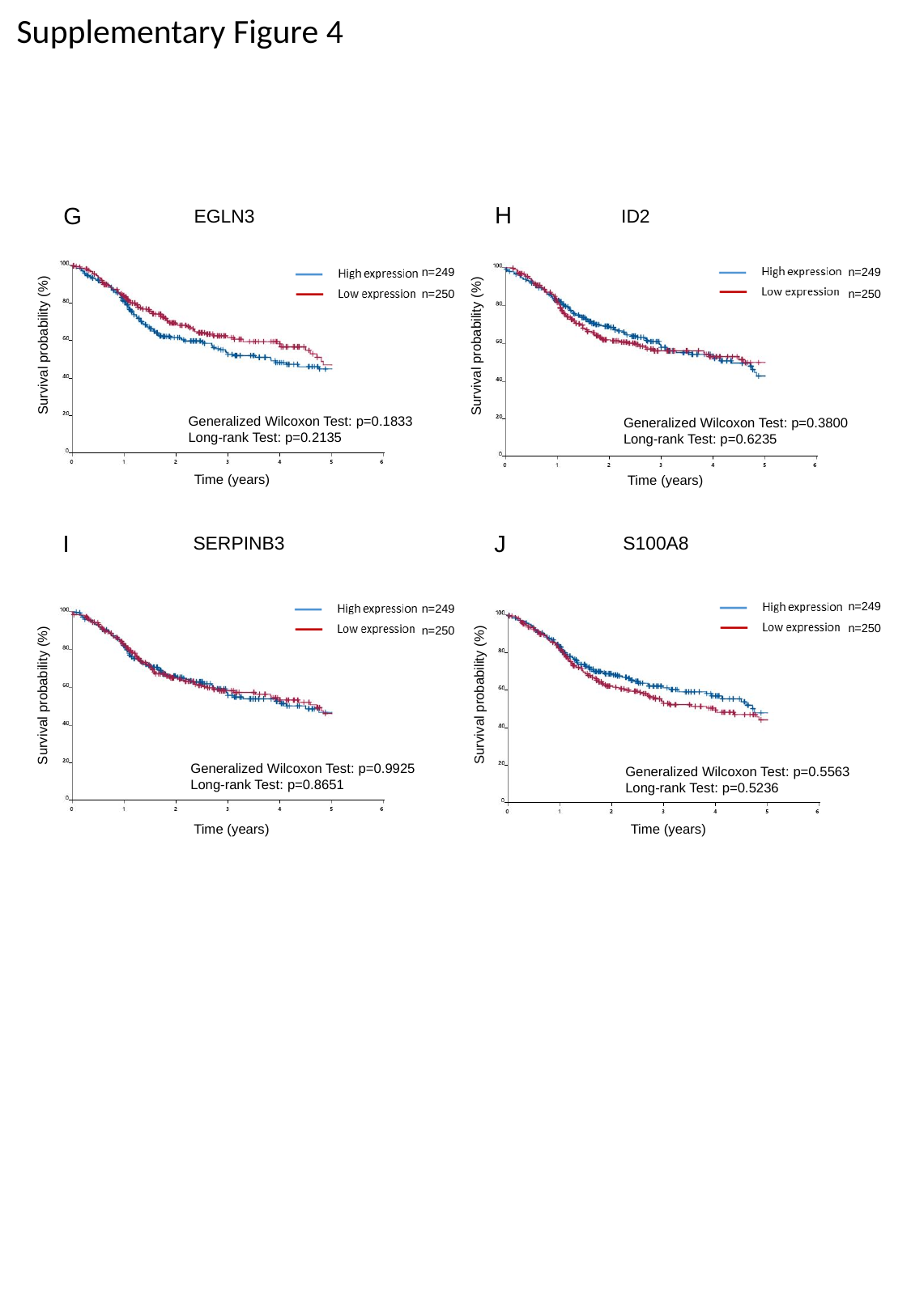

Supplementary Figure 4
H
G
EGLN3
ID2
n=249
n=250
n=249
n=250
Survival probability (%)
Survival probability (%)
Generalized Wilcoxon Test: p=0.1833
Long-rank Test: p=0.2135
Generalized Wilcoxon Test: p=0.3800
Long-rank Test: p=0.6235
Time (years)
Time (years)
J
I
SERPINB3
S100A8
n=249
n=250
n=249
n=250
Survival probability (%)
Survival probability (%)
Generalized Wilcoxon Test: p=0.9925
Long-rank Test: p=0.8651
Generalized Wilcoxon Test: p=0.5563
Long-rank Test: p=0.5236
Time (years)
Time (years)
