# Supplementary material for: Prognostic association of starvation-induced gene expression in head and neck cancer
Source: Sci Rep. 2021 Sep 27;11:19130. doi: 10.1038/s41598-021-98544-1 (PMC8476550; doi:10.1038/s41598-021-98544-1)
Supplement: Supplementary file 2 — Supplementary Information 2. [file 41598_2021_98544_MOESM2_ESM.docx]

**Table S1. Summary of RNA-seq**

| Sample name | #Reads |  | Mapping rate | No. of detection genes | No. of genes with >0 counts | No. of filter-passing genes |
| --- | --- | --- | --- | --- | --- | --- |
| control 0h-1 | 55997132 |  | 94.5% | 26256 | 17966 | 6363 |
| control 0h-2 | 42485264 |  | 93.4% | 26256 | 17509 | 6363 |
| control 2h | 42644844 |  | 92.5% | 26256 | 17477 | 6363 |
| control 24h | 44574770 |  | 95.2% | 26256 | 17488 | 6363 |
